# High-Frequency Jet Ventilation During Cryoablation of Small Renal Tumours

**DOI:** 10.1007/s00270-018-1921-4

**Published:** 2018-03-07

**Authors:** Thea Buchan, Miles Walkden, Kathryn Jenkins, Pervez Sultan, Steve Bandula

**Affiliations:** 1 Imaging Department, University College London Hospital, 235 Euston Road, London, NW1 2BU UK; 2 Department of Anaesthesia, University College London Hospital, 235 Euston Road, London, NW1 2BU UK; 3 Centre for Medical Imaging, University College London, 250 Euston Road, London, NW1 2PG UK

**Keywords:** Kidney, Cancer, Cryoablation, Ventilation, Computed Tomography

## Abstract

**Aim:**

To evaluate the effect of high-frequency jet ventilation (HFJV) in place of standard intermittent positive-pressure ventilation (IPPV) on procedure duration, patient radiation dose, complication rates, and outcomes during CT-guided cryoablation of small renal tumours.

**Materials and Methods:**

One hundred consecutive CT-guided cryoablation procedures to treat small renal tumours under general anaesthesia were evaluated—50 with standard IPPV and 50 after the introduction of HFJV as standard practice. Anaesthesia and procedural times, ionising radiation dose, complications, and 1-month post-treatment outcomes were collected.

**Results:**

HFJV was feasible and safe in all cases. Mean procedure time and total anaesthetic time were shorter with HFJV (*p *= <0.0001). The number of required CT acquisitions (*p *= 0.0002) and total procedure patient radiation dose (*p *= 0.0027) were also lower in the HFJV group compared with the IPPV group. There were a total of four complications of Clavien–Dindo classification 3 or above—three in the IPPV group and one in the HFJV group. At 1-month follow-up, two cases (both in the IPPV group) demonstrated subtotal treatment. Both cases were subsequently successfully retreated with cryoablation.

**Conclusion:**

By reducing target tumour motion during CT-guided renal cryoablation, HFJV can reduce procedure times and exposure to ionising radiation. HFJV provides an important adjunct to complex image-guided interventions, with potential to improve safety and treatment outcomes.

## Introduction

Modern management of small renal masses has moved towards nephron-sparing techniques—that include partial nephrectomy (PN) and image-guided ablation (IGA). IGA allows minimally invasive tumour destruction with significantly reduced patient impact [1], preserved renal function [2], and oncological outcomes that are equivalent to surgical resection [3].

Minimising rates of local recurrence following IGA requires diligent technique to ensure ablative energy is delivered precisely to the tumour with an appropriate margin. Cryoablation (CYA) has become the technique of choice, with multiple probes carefully positioned simultaneously within the tumour to produce a conformal treatment zone or ‘iceball’ that can be visually confirmed on intra-operative CT or MRI.

Despite immobilisation with general anaesthesia, traditional intermittent positive-pressure ventilation (IPPV) causes significant abdominal organ motion making tumour targeting more difficult [4]. Probes must be inserted between respiratory cycles or during short periods of apnoea—often requiring additional image acquisitions and extending procedure length.

High-frequency jet ventilation (HFJV) delivers continuous small tidal volume ventilation at high frequency resulting in a near stationary diaphragm and minimal target organ motion [5–8]. Several studies have reported a reduction in technical difficulty associated with probe placement, procedure time, and patient radiation dose during image-guided interventions and single-probe thermal ablation of metastatic tumours in the liver and lung [9–11]. HFJV has the potential to simplify renal tumour cryoablation, where multiple simultaneous probe placements are required, and where treatment margins must be adequate but not excessive to preserve renal function [12].

The aim of this study was to evaluate the effect of HFJV in place of standard IPPV on procedure duration, patient radiation dose, and complication rates during CT-guided cryoablation of small renal tumours.

## Methods

### Study Design

This retrospective review was undertaken at a single tertiary interventional oncology (IO) centre with prior experience of over 150 renal tumour cryoablation cases. The IO team had also completed eight CT-guided ablation procedures (five lung and three liver) using HFJV before this study. One hundred consecutive patients undergoing CT-guided cryoablation of a small renal tumour under general anaesthesia between December 2015 and January 2017 were evaluated—50 with standard IPPV and 50 after the introduction of HFJV as standard practice at this centre. The study was reviewed by the University College London and University College London Hospitals joint research and development office and considered to be service evaluation exempt from research ethics committee approval.

The decision to treat with CYA was made at the local renal cancer network multidisciplinary team meeting. All patients gave fully informed consent, and in all cases, treatment was performed with curative intent. The primary outcome for this study was procedure duration. Secondary outcomes included: probe placement time, patient radiation dose (represented by dose length product (DLP) generated automatically at the scanner console), number of CT acquisitions (fluoroscopic acquisitions required for needle insertion), and complication rates (anaesthetic, medical or surgical score according to the Clavien–Dindo scale) following CT-guided cryoablation of small renal tumours.

### Anaesthetic Protocol

All patients were subject to anaesthetic pre-assessment and were classified per the physical status classification system of the American Society of Anaesthesiologists (ASA). General anaesthesia was induced by one of two anaesthetists with 1–2 mg midazolam, followed by a total intravenous anaesthesia target controlled intravenous infusion of propofol (maintenance range 1.8–6 mcg/ml) and remifentanil (0.2–8 ng/ml). After muscle relaxation, patients were intubated orally and a reinforced endotracheal tube (ETT) inserted. The patients were then positioned in the CT scanner to allow optimal tumour access in either a prone or lateral position.

All patients were initially ventilated with conventional volume- or pressure-controlled intermittent positive-pressure ventilation, via a standard circle anaesthetic breathing system using a Datex Aestiva anaesthetic machine. For those patients in the HFJV group, a jet swivel adaptor (Acutronic Medical Systems) was introduced into the circuit between the patient’s cuffed oral ETT and the anaesthetic breathing circuit.

HFJV was commenced at 100–120 breaths per minute, with a driving pressure (DP) of 1.0 bar. DP was adjusted to maintain the end-tidal carbon dioxide between 4.5 and 5.5 kPa using intermittent checks measured through IPPV via the anaesthetic machine capnography device (GE Healthcare gas module E-CAi0-00 (Helsinki, Finland)).

Once CYA treatment was complete, the patients were ventilated conventionally until awake.

### CT Protocol

All renal cryoablation procedures were performed in a dedicated interventional CT suite (Toshiba Prime, Toshiba Medical Systems, Japan, 2014) following the standard CT-guided renal cryoablation protocol at our institution. Patients were positioned in either the prone or lateral position to allow optimal target tumour access—confirmed using a low-dose unenhanced positioning CT scan. A dual (arterial and venous)-phase CT scan of the kidneys was performed to plan probe placement and adjunctive hydro- or pneumo-dissection.

The CT fluoroscopic package in intermittent biopsy mode was used for guidance of cryoprobe placement (Galil Medical System, Minneapolis, USA). Each acquisition comprised a volume (100 kV, 30 mA with 0.5 s gantry rotation) reconstructed into three congruent 4 mm slices. Final cryoprobe placement was confirmed with a low-dose scan volume of the target organ before commencement of treatment with a double freeze (10 min) and thaw (6 min passive and 2 min active) protocol. During each cycle, low-dose scans of the target organ were obtained at 5 and 10 min to monitor the ice ball formation. A final low-dose post-procedure scan was performed following cryoprobe removal as a safe check to look for immediate complications such as haemorrhage or pneumo-thorax. Hydrodissection was administered when required using a 21-gauge spinal needle to infuse sterile saline containing Omnipaque at a ratio of 20 per 1000 ml of saline. Hydrodissection needles were left in situ during treatment cycles.

All helical scans used the following standard parameters: 100 kV tube potential, automatic tube current modulation with a range of 40–180 mA, 0.5 s gantry rotation, 0.5 detector collimation, a pitch factor of 0.813, a helical pitch of 65, and iterative reconstruction with 1 mm slice thickness.

For each procedure, data related to procedure duration, patient-ionising radiation dose, technical difficulty, and complications were collected. Technical difficulty was assessed through the surrogate measures of probe placement time and total number of CT acquisitions for probe placement. Total ionising radiation dose was recorded as dose length product (DLP) generated automatically at the scanner console.

Anaesthetic induction time was defined as the period from initiating induction of anaesthesia to commencement of the positioning scan, and total procedure time was defined as time from positioning scan to the final treatment confirmation scan. The number of cryoprobes, hydro- and pneumo-dissection needles used, number of biopsy-mode CT volume acquisitions, and total time required to place the probes and needles were also recorded.

### Patient Follow-Up

All patients were admitted overnight for observation and if standard hospital discharge criteria met, discharged the following morning. Patients were reviewed in an outpatient clinic at 1 month post-treatment with renal contrast-enhanced CT. Treatment was considered successful if at 1 month post-procedure, the ablation zone encompassed the target tumour with a margin greater than 5 mm in all planes, and with no tumour enhancement [13]. All complications within 30 days of treatment were recorded and scored using the Clavien–Dindo classification system.

### Statistical Analysis

Data between IPPV and HFJV groups were compared using unpaired *t* tests. A *p* value of 0.05 or lower was considered statistically significant. IPPV and HFJV groups were assessed for normal distribution using Kolmogorov–Smirnov tests. Nonparametric data between groups were compared using Mann–Whitney *U* test. All statistical tests were carried out using PRISM (GraphPad Software, San Diego, Calif).

## Results

During the review period, 100 renal tumours were treated by CT-guided cryoablation in 100 separate treatment sessions involving 95 patients. Fifty treatments were performed using IPPV and 50 using HFJV. No patients were excluded from the analysis, and patient records and data were available for all cases. None of the patients intended for HFJV required a switch in ventilation technique to IPPV. Treatment was successfully completed (technical success) in all cases at the time of procedure. However, at 1-month follow-up, subtotal treatment was found in two patients in the IPPV group, requiring retreatment of cryoablation. None of the patients treated using HFJV required repeat treatment.

**Fig. 1:**
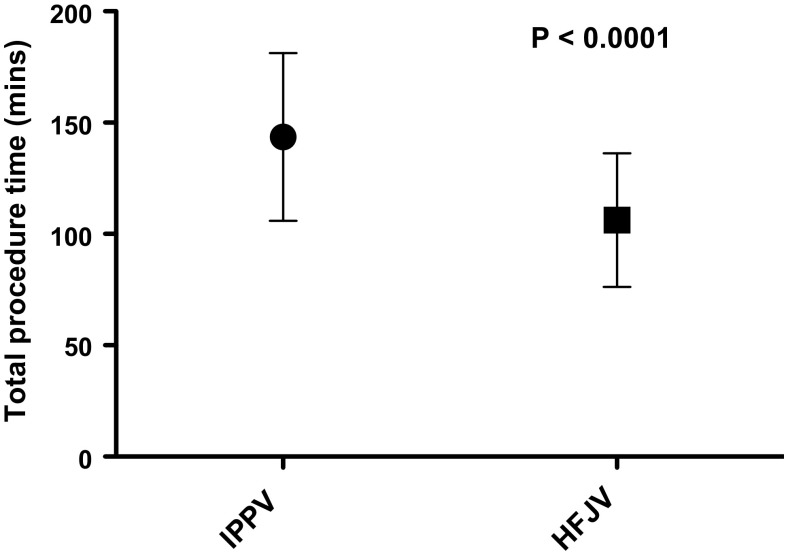
Box-and-whisker plot showing total procedure time (mean and standard deviation) in minutes for the two ventilation groups

**Fig. 2:**
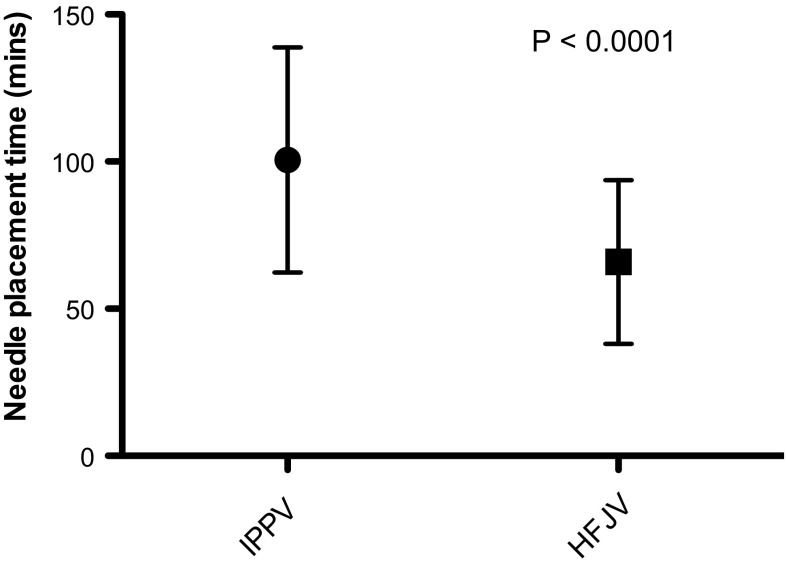
Box-and-whisker plot showing needle placement time (mean and standard deviation) in minutes for the two ventilation groups

**Fig. 3:**
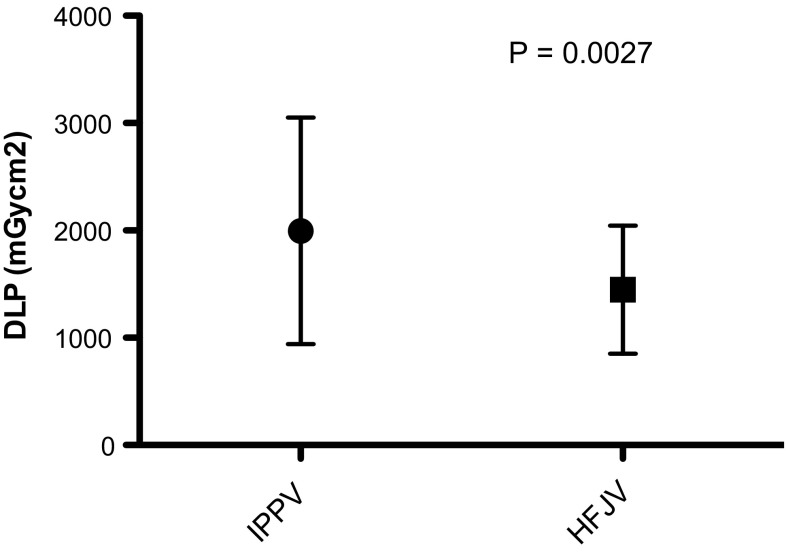
Box-and-whisker plot showing total procedure dose (mean and standard deviation) as dose length product for the two ventilation groups

Patient demographics and tumour characteristics are summarised in Tables 1 and 2, with comparable characteristics and ASA classification between groups. Table 3 summarises details of treatment sessions including patient position, anaesthetic induction time, number of cryoprobes placed, and the use of hydro- and pneumo-dissection. Comparison between groups showed no statistically significant difference in these variables. Mean total procedure time was 144 min (SD 37.32) for IPPV versus 106 min (SD 29.69) for HFJV (*p *= <0.0001) (Fig. 1), and mean needle placement time was 100 min (SD 38.24) for IPPV versus 66 min (SD 27.83) for HFJV (*p *= <0.0001) (Fig. 2). The number of CT acquisitions required for needle placement was 78.8 (SD 29.02) for IPPV versus 59.2 (SD 21.98) for HFJV (*p *= 0.0002) (Table 4), and total procedure patient radiation dose 1975 mGycm2 (SD 1055) for IPPV versus 1449 mGycm2 (SD 597.4) for HFJV (*p *= 0.0027) (Fig. 3).

**Table 1:** Patient demographic details—mean (range)

	IPPV group	HFJV group
Number of procedures	50	50
*Sex*		
Male	35	39
Female	15	11
Age (years)	64 (28–86)	69 (41–85)
Weight (Kg)	91 (73–138)	85 (65–128)
Height (metres)	1.70 (1.56–1.91)	169 (1.5–1.87)
Body mass index (kg/m2)	31.4 (22.2–43)	29.5 (21.1–42.2)
ASA	2.54	2.68

**Table 2:** Baseline tumour characteristics

	IPPV	HFJV	*p* value
PADUA score	7.2	7.41	
Size (mm)	25.63	28.74	0.1322
*Histology*			
Clear cell	31	35	
Chromophobe	3	5	
Papillary	12	8	
Spindle cell	1	0	
Oncocytoma	1	0	
Not biopsied	2	2

**Table 3:** Comparison of procedural details

	IPPV	HFJV	*p* value
*Patient position*			
Prone	38	31	–
Left lateral	5	9	–
Right lateral	7	10	–
Induction time (total anaesthesia time minus procedure time)	30 min 4 s	31 min	0.2788
Number of probes	3.922	4.24	0.3427
Hydro or pneumo-dissection	25	23	–

There were no complications or delays to discharge attributable to ventilation technique. There were no deaths within the 30-day follow-up period. There were 17 complications, four of which with Clavien–Dindo (CD) score of 3 or above. These were one case of transient brachial plexus injury (CD3) which resolved without intervention; one urine leak (CD3b), managed conservatively with insertion of a ureteric stent; one case of haematuria (CD3a), requiring bladder irrigation via a three-way catheter; and one patient developed a right-sided pulmonary embolus (CD4a) requiring an ITU admission and delaying discharge by 15 days.

**Table 4:** Comparison of time/dose/difficulty

	IPPV	HFJV	*p* value
Total procedure time (minutes)	142.4	105.8	< 0.0001
Needle placement time (time to achieve probe positions—minutes)	100.2	66.2	< 0.0001
Number of acquisitions for probe placement	78.8	59.2	0.0002
Patient dose collected as dose length product (mGycm2)	1975	1449	0.0027

At 1-month follow-up imaging, two cases (both in the IPPV group) demonstrated subtotal treatment. Both cases were subsequently successfully retreated with cryoablation.

## Discussion

Image-guided ablation has established itself within the toolbox of modern oncology. For small renal tumours, advances in technique, image guidance, and ablation technology allow image-guided ablation (IGA) with equivalent oncological outcomes to surgery [3], and IGA has moved away from being used only in high-risk surgical groups to an effective primary therapy for localised cancer [13, 14]. Technique development is now focused on achieving more robust, consistent, and cost-effective delivery of treatment [15], with improved tumour targeting a crucial factor in achieving these aims.

By reducing target organ motion, HFJV has potential to reduce technical difficulty associated with treatment probe placement in solid organs such as the liver and kidney, thereby improving tumour targeting and accuracy. Technical difficulty is problematic to quantify, but logical surrogate measures related to procedure duration and required level of image guidance can be evaluated.

In this retrospective review, we demonstrate that HFJV can be safely implemented and produces a significant reduction in procedure time, number of required CT acquisitions, and ionising radiation dose for patients undergoing cryoablation of small renal tumours, with no effect on complication rate or length of inpatient stay.

CT-guided cryoablation procedures frequently place significant pressure on CT capacity due in part to the length of procedure. The 26% reduction in total procedure time demonstrated here will have cost and resource benefits to interventional oncology departments, justifying the investment in additional equipment and training that may be required to perform HFJV. A significant reduction in procedure and anaesthetic time will reduce anaesthetic risk and allow more rapid recovery [16, 17]. Reduced anaesthetic risk will also widen access to patients who may have comorbidities and are unable to undergo other treatments.

Complex interventions require multiple CT acquisitions. Modern scanners implement a range of dose sparing techniques such as dose modulation and iterative reconstruction; however, dose to the patient and operator during these procedures is significant. With the current trend of increasing numbers of IGA treatments being performed, methods that reduce dose exposure to patient and operator are particularly important. By reducing the number of acquisitions required to place the probes (24%), HFJV has a direct effect on the ionising radiation dose to the patient. We report a 27% reduction in patient radiation dose (DLP—mGycm^2^) for procedures performed under HFJV compared with IPPV. The patient dose reduction achieved is significant (an increasing number of whom are < 50 years), and although not measured here, would likely lead to a reduced scatter dose to the operator. This dose reduction is, however, less than the 54% reduction reported by Chung et al., achieved with HFJV during thermal ablation of lung tumours, which may reflect the higher number of set helical acquisitions (e.g., position checking and treatment monitoring helical scans) within the renal tumour treatment protocol.

We used needle placement time and number of CT acquisitions to evaluate the ability of HFJV to reduce the technical difficulty of CT-guided renal cryoablation by reducing ventilatory motion. Needle placement time fell from 100 to 66 min between groups (*p *= <0.0001), and number of CT acquisitions from 78.8 to 59.2 between groups (*p *= <0.0002)—giving a clear indication of the effect of HFJV. Factors other than motion may affect needle placement time, including depth of tumour from the skin, tumour endophycity, and patient position. Patient BMI, position, and tumour PADUA score were well matched between groups (Table 2), minimising this potential bias. Although we included placement of dissection needles in the evaluation, we did not correct for variation in the time and CT acquisitions required to achieve satisfactory hydro- or pneumo-dissection.

Importantly, we demonstrate that in a representative patient group, HFJV can be applied ubiquitously without the need for conversion to IPPV and that establishing anaesthesia with HFJV was safe and took no longer. The requirement for the anaesthetic team to undergo additional training to be familiar and confident with the use of HFJV is recognised [9], and special arrangements were made during the evaluation period to ensure specific anaesthetist staff were available. However, despite the benefits described, limitations in training and staff availability may limit use with IGA.

The use of HFJV in the prone position may be unfamiliar to many anaesthetic teams. Prone ventilation is used widely in the intensive care environment to optimise PaO2: FiO2 ratio, with no increase in risk of barotrauma, CO_2_ clearance, or ability to deliver tidal volumes [18]. These risks are not thought to be altered by the use of HFJV in the prone position, but this has not been specifically investigated. Concerns associated with a prone position are dislodging tubes and lines; abdominal discomfort and compartment syndrome in obese patients; and nerve damage associated with arm positioning and pressure point damage rather than issues associated with the mode of ventilation.

Patient age was also not a limitation to HFJV. Out of 95 patients in this study, 72 were aged over 60 years. We experienced no issues with hypercapnoea, acidosis, or tube placement that required intervention or deviation from our predetermined protocol. Severe COPD has been considered to be a relative contraindication to HFJV, due to the potential risk of gas trapping and barotrauma in this population; however, in the range of lung function normally acceptable for ablation under general anaesthesia at our institution (i.e., an FEV1 > 1L), this has not thus far been  a significant concern. Further studies are however required to specifically explore the acceptability of this technique in patients with severe cardiorespiratory disease to determine whether any patient cohorts should be precluded from HFJV in the prone and lateral positions.

There were several further limitations to this study. The review was conducted retrospectively and without randomisation and therefore potentially subject to several sources of bias. However, the compared groups did consist of consecutively treated patients, with good matching of key patient and tumour characteristics and no missing data—thus allowing meaningful comparison between the HFJV and the IPPV control group. Although sufficient to demonstrate change in the endpoints assessed here, the case numbers were relatively small, and not sufficient to probe differences in technical success or complication rate.

The post-treatment follow-up period was also short (1 month) and insufficient to demonstrate any effect on treatment efficacy. We hypothesise that reduced tumour motion and more accurate targeting would lead to higher treatment efficacy and improved oncological outcomes, but this requires future investigation.

## Conclusion

By reducing target tumour motion during CT-guided renal cryoablation, HFJV can reduce the technical difficulty associated with probe insertion—leading to reduced procedure times and exposure to ionising radiation. HFJV provides an important adjunct to complex image-guided interventions, with potential to improve safety, treatment outcomes and extend the scope of IGA therapies; however, further studies are required to evaluate these effects.
